# Perception and Attitude of Turkish Gastroenterologists Toward Obesity: A Nationwide Survey Conducted by the Obesity Study Group of the Turkish Gastroenterology Association

**DOI:** 10.5152/tjg.2024.22733

**Published:** 2024-03-01

**Authors:** Mustafa Tahtacı, Emre Yıldırım, Enver Üçbilek, İlker Şen, Gülden Bilican, Zahide Şimşek, Süheyla Ayça Gümüş, Murat Aladağ, Murat Kıyıcı, Mehmet Cindoruk, Tarkan Karakan

**Affiliations:** 1 Department of Gastroenterology, Ankara Yıldırım Beyazıt University Faculty of Medicine, Ankara, Turkey; 2 Depatment of Gastroenterology, Memorial Hospital, İstanbul, Turkey; 3 Department of Gastroenterology, Mersin University Faculty of Medicine, Mersin, Turkey; 4 Department of Gastroentrology, Şişli Training and Research Hospital, İstanbul, Turkey; 5 Department of Gastroentrology, Gazi University Faculty of Medicine, Ankara, Turkey; 6 Department of Gastroentrology, Health Sciences University, Dışkapı Yıldırım Beyazıt Education and Training Hospital, Ankara, Turkey; 7 Department of Gastroenterology, Ankara Bilkent City Hospital, Ankara, Turkey; 8 Department of Gastroentrology, Malatya Turgut Özal University Faculty of Medicine, Malatya, Turkey; 9 Department of Gastroenterology, Uludağ University Faculty of Medicine, Bursa, Turkey

**Keywords:** Obesity, gastroenterology, survey

## Abstract

**Background/Aims:**

Gastroenterologists have an important role in the treatment and management of comorbidities related to obesity. Assessment of gastroenterologists’ perception and attitude toward obesity was aimed in this study.

**Materials and Methods:**

Survey questions were determined for the study. An online questionnaire was prepared afterward. Participants were invited via e-mail by providing them with information about the study. It was ensured that those who accepted the study could access the questionnaire form with the relevant link. Participants who answered all questions were included in the study.

**Results:**

Totally 117 gastroenterologists were included in the study. The proportion of gastroenterologists who thought that obesity complicates the management of gastroenterological diseases and those who thought obesity as a factor that negatively affects the prognosis of gastroenterological diseases was determined as 88.9% and 95.7%, respectively. Among the obese patients, the proportion of those who applied diet therapy, exercise, pharmacotherapy, and endoscopic methods was 94%, 91.5%, 35%, and 37.6%, respectively. The rates of intragastric balloon therapy and intragastric botulinum toxin A injection were 30% and 21.4%, respectively. The proportion of those who agreed that obese patients lost a significant amount of weight with the treatment methods applied was 47.2%. The proportion of participants who agreed that long-term maintenance of weight loss was impossible for most obese patients was 59.8%.

**Conclusion:**

To our knowledge, this is the first study that evaluates the perception and attitude of gastroenterologists toward obesity. Our study results show that gastroenterologists think that obesity is a chronic disease and that gastroenterologists should be involved in management of obesity.

**Main Points**

- Management of obesity requires a multidisciplinary approach.
- Gastroenterologists think that obesity is a chronic disease and that gastroenterologists should take part in the treatment of obesity.
- The most frequently preferred endoscopic treatment method by gastroenterologists was the intragastric balloon therapy (IGBT).

## Introduction

Obesity is a chronic complex disease that results in increasing long-term complications and reducing life expectancy.^1^ With increasing average body mass index in children and adolescents since the last 40 years, obesity has become a global pandemic disease.^2^

Obesity treatment is composed of behavioral changes, psychological, pharmacological, and bariatric treatment approaches.^3^ Bariatric endoscopic treatments compared with bariatric surgery are less invasive options for weight loss in obese patients.^4^ Despite the variety of options, obesity treatments have low success rates in long-term follow-up.^5^ Although patient-related factors are an important cause of failure of obesity treatments, physician-related factors can also contribute to it. In literature there is strong evidence about physicians' inadequate counsellation of obese patients and physician attitudes may be one reason for this failure.^6^

Obesity is an important risk factor for some diseases of the gastrointestinal (GI) system.^7^ Obesity-related GI disorders are more common and have earlier symptoms as compared with type 2 diabetes mellitus and cardiovascular diseases; thus, gastroenterologists have the opportunity to provide an early and effective treatment for obesity.^8^

Gastroenterologists are an important part of the multidisciplinary team of obesity with their ability to use nutrition and endometabolic bariatric methods effectively. On the other hand, there no study in the literature evaluating perceptions and attitudes of gastroenterologists toward obesity. Assessment of perceptions and attitudes of gastroenterologists toward obesity was aimed in this study.

## Materials and Methods

### Study Population

The Obesity Study Group members of the Turkish Gastroenterology Association designed a questionnaire consisting of 21 items in order to evaluate attitude, perception, and awareness of gastroenterologists toward obesity. Some of the survey questions were adapted from surveys in various studies in the literature.^9,10^ Gastroenterology specialists and fellowship members registered with the Turkish Gastroenterology Association were included in the study. An online survey with 21 items was distributed using Google Forms. An invitation to participate in the survey, containing information about the study, was sent via e-mail to 948 gastroenterology specialists and 169 fellowship members registered with the Turkish Gastroenterology Association. Physicians who answered all questions were included in the study. The survey was sent to gastroenterologists 2 times via e-mail. In order to increase participation rate of the survey, support was requested from gastroenterologists in the Obesity Study Group panels at national scientific meetings. Ethics committee approval was received for this study from the Ethics Committee of Gazi University Faculty of Medicine (Date: November 4, 2021, Approval Number: E206097). Participation in the survey was completely voluntary. 

### Statistical Analysis

Preliminary analyses were completed for frequencies, means, SD, and percentages where applicable. Categorical variables were analyzed by chi-square test. The statistical significance was defined as *P* < .05. All statistical analyses were conducted using the Statistical Package for the Social Sciences for Windows version 17.0 (SPSS Inc., Chicago, Ill, USA).

## Results

Totally 117 participants who answered the survey questions were included in the study. 31.6% of the participants were female gastroenterologists. 37.6% of the participants in the study were between the ages of 40 and 50 years. The proportion of those with more than 20 years of gastroenterology professional experience was determined as 19.7%. Demographic characteristics of the gastroenterologists participating in the study are given in Table 1.

About 67.5% of the participants stated that they evaluated the body mass index (BMI) of the patients who applied to the outpatient clinics. Participants stated that 72.6% of the patients they followed were overweight and 22.3% were obese according to BMI. It was determined that 94.1% of the participants agreed and strongly agreed that obesity is a chronic disease. It was determined that 77.8% of the participants agreed and strongly agreed that obese patients were difficult to treat compared to normal-weight patients. Percentage of participants who did not have negative reactions to the appearance of obese patients was found to be 56.4%. The proportion of those who thought that obesity complicates the management of GI diseases and those who thought obesity as a factor that negatively affects the prognosis of GI diseases was determined as 88.9% and 95.7%, respectively. The proportion of those who believed that obese patients should be educated about the health risks of obesity was 98.3%, whereas the proportion of those who participated in the involvement of gastroenterologists in the treatment of obesity was found to be 88%. The proportion of those who agreed that obese patients lost a significant amount of weight with the applied treatment methods was 47.2%. The proportion of those who agreed that long-term maintenance of weight loss was impossible for most obese patients was 59.8%. The perception of gastroenterologists toward obesity is given in Table 2.

The proportion of those who agreed that physical inactivity, overeating, and high-fat diet were important or very important factors in the etiology of obesity was 91.4%, 92.3%, and 80.4%, respectively. Conditions that the participants considered as the cause of obesity are given in Table 3.

About 91.5% of the gastroenterologists in the study stated that obese patients were making an effort for weight control. Factors preventing gastroenterologists from participating in weight management of obese patients were lack of interest to obesity treatment in 20.5%, lack of sufficient time in 43.6%, and lack of effective treatment options in 24.9%. The factors preventing obese patients being involved in weight control are given in Table 4.

Among the obese patients, the proportion of those who applied diet therapy, exercise, pharmacotherapy, and endoscopic methods was 94%, 91.5%, 35%, and 37.6%, respectively. However, 1.7% did not apply any treatment. Intragastric balloon therapy (IGBT) was the most common endoscopic method and was applied by 30%. Intragastric balloon therapy was followed by intragastric botulinum toxin A (BTA) injection (21.4%) and endoscopic sleeve gastroplasty (1.7%). The most common complications of bariatric surgery reported by the participants were leak and fistula (85.3%), followed by bleeding (7.3%) and stricture (5.9%).

The proportion of participants who agreed that obesity treatment was important or very important in the management of gastroesophageal reflux disease (GERD), functional dyspepsia, irritable bowel syndrome (IBS), inflammatory bowel disease (IBD), nonalcoholic fatty liver disease (NAFLD), and GI cancers was 63.3%, 44.4%, 34.2%, 14.6%, 82,1%, and 57.2%, respectively (Table 5).

## Discussion

Obesity is defined as health-threatening abnormal and excess fat accumulation.^11^ Ectopic and excess body fat causes cardiometabolic diseases and increased cancer risk via a significant source of adipokine inflammatory mediators.^3^ Obesity causes risk of many diseases such as GERD, NAFLD, gall bladder stones, acute pancreatitis, and GI cancers.^7,8^ Gastroenterologists commonly encounter obesity-related GI disorders. Perception and attitudes of gastroenterologists toward obesity were evaluated in this study.

Although obesity was first included in International Classification of Diseases in 1948, World Health Organization defined obesity as a chronic disease in 1997. In our study, a significant part of the participants agreed that obesity is a chronic disease. Classifying obesity as a disease may help to decrease being stamped, may provide awareness against fake weight loss treatments, and may expand research about the pathophysiology of weight gain.^12^ Weight prejudice and stigma can hinder the care provided to patients with obesity.^13^ Although a significant part of the participants in our study stated that obese patients are difficult to treat compared to normal-weight patients and that obesity complicates the management of GI diseases, they state that they do not have negative reactions to the appearance of obese patients. Obesity is not only a risk factor for the occurrence of some GI disorders but may also adversely affect clinical outcomes by reducing the response of GI disorders to specific treatments.^14^ Similarly, a significant part of the participants thought that obesity was a negative prognostic factor of GI disorders. Providing weight loss counseling to obese patients helps long-term achievement of clinically significant weight loss in approximately one-third of obese patients.^15^ A significant portion of the participants in our study believed that obese patients should be educated about the health risks of obesity. Gastroenterologists may play an important role in multidisciplinary management of obesity.^8^ In the study, a significant part of the participants believed that gastroenterologists should involve in the treatment of obesity and stated that they provided a significant amount of weight loss with the treatment methods they applied to obese patients. However, most of the participants thought that long-term maintenance of weight loss in obese patients was impossible. A significant part of obese patients regain the weight they lost within 5 years.^16^ The inability of weight loss sustainability in obese patients is an important problem.

It has been suggested that many factors may play a role in the development of obesity.^17^ In the study evaluating the perceptions and attitudes of primary care physicians to the treatment of obesity, it is stated that physical inactivity, overeating, and a high-fat diet are the most important causes of obesity.^9^ Similarly, the 2 most important factors seen as the cause of obesity in our study were physical inactivity and overeating. High-fat diet, genetic factors, poor nutritional knowledge, psychological problems, lack of willpower, metabolic disorder, endocrine disorder, educational status, and genetic disorders were other important causes found by the participants in the study. Income status and gut microbiota were among the reasons that were considered insignificant by the study participants. It is stated that there is an inverse causality relationship between obesity and income status and more detailed evaluations are needed for understanding this inverse relationship.^18^ Although the participants in our study did not consider the gut microbiota as one of the causes of obesity, the gut microbiota has been identified as a potential factor in the pathophysiology of both obesity and related metabolic disorders and has been suggested as a new way to treat obesity.^19^

While it was stated that the most important factor preventing gastroenterologists from being involved in the weight management of obese patients was the lack of interest in the obesity treatment, the lack of sufficient time and the ineffectiveness of treatment options were the factors that were found to be unimportant. However, the rate of gastroenterologists not treating obese patients was found to be quite low. Diet and exercise are first-line treatment options in obesity. Endoscopic bariatric treatments are one of the most interesting treatment modalities in obesity. Intragastric balloon therapy was the mostly applied endoscopic method by the gastroenterologists who participated in the study. Among endoscopic bariatric treatments, IGBT is a treatment option that has been used for many years. Intragastric balloon therapy with moderate- to high-intensity lifestyle therapy as a weight-loss intervention over lifestyle interventions alone is suggested in American Gastroenterological Association Guideline for obesity.^20^

Intragastric BTA injection in obesity treatment is controversial. Intragastric BTA injection is the second most frequently preferred endoscopic method for obesity treatment by the gastroenterologists who participated in the study. In a meta-analysis evaluating recent randomized controlled trials, intragastric BTA injection was found effective in obesity treatment.^21^ Although endoscopic sleeve gastroplasty was a method that was applied at a very low rate among endoscopic treatments, other treatments such as gastric aspiration and duodenal mucosal resurfacing were the methods that were not preferred. The reasons why the participants did not prefer these methods can be listed as follows: these methods are not easily accessible and incur a high cost or there is a lack of ample number of properly designed randomized controlled studies on the effectiveness of these methods.

Bariatric surgery may maintain sustainable weight loss in obesity treatment.^22^ Although improvements in bariatric surgery complications are not rare,^23^ bariatric surgery complications vary between types of surgical procedures. The most commonly observed complications of bariatric surgery by the participants were leak and fistula, and they are the main complications that increase postoperative early period morbidity and mortality.^24^ However, endoscopic methods are effective in the treatment of leak and fistula.^25^ Bleeding and stricture were less common complications observed by the participants; the rareness of these complications may be attributed to the type of bariatric surgery opted for in participants’ centers.

Obesity plays a role in development of many diseases. The most important target in obesity treatment is prevention of obesity-related multimorbidities.^26^ Obesity treatment has a positive effect not only on the prevention of diseases but also on the course of diseases. While obesity is directly effective in the development of some diseases in terms of gastroenterology, it is an important risk factor for some diseases.^8^ In our study, participants considered obesity to be a significant risk factor for NAFLD, functional dyspepsia, and GI cancers; however, this consideration was not statistically significant for IBS. Although it has been suggested that obesity may play a role in the etiopathogenesis of IBS, the results of the studies are inconsistent.^14,27,28^ A significant portion of the study participants stated that they found the effect of obesity treatment on IBD less important. The effectiveness of obesity treatment for IBD is controversial. On the other hand, it is stated that a considerable portion of IBD patients are obese and obesity may have a negative effect on the course of IBD.^29^ There is a need for studies on the relationship between obesity and IBD in which larger populations should be included and additional factors such as microbiota, diet patterns, and the effect of weight loss on the course of the disease should be evaluated.^30^

Our study has several limitations. First, the survey did not include some detailed questions, such as the institutions where the gastroenterologists work, their participation in the Obesity Study Group, the level of education of gastroenterologists about endoscopic and specific medical treatments of obesity, the number of obese patients followed, the number of patients who received specific medical and endoscopic treatments in their clinic within a year, complications of the treatment methods, collaboration with other disciplines such as surgery and endocrinology, and the presence of a specific polyclinic for obesity in their centers. Secondly, the distribution of the years of professional experience of the gastroenterologists included in the study is not homogeneous. Thirdly, it is not known how meticulous the participants were in answering the questions in the online survey studies, and fourth, some participants may have exhibited a biased attitude toward obesity. Another limitation of the study was that there was no question about the centers where the gastroenterologists worked. The type of centers where the participants work can have a role in the effectiveness of their treatment choices. Another limitation of the study is the low participation rate of gastroenterologists in the survey. But poor participation of physicians in surveys is an international problem in health sciences.

In conclusion, our study shows that gastroenterologists think that obesity is a chronic disease and that gastroenterologists should take part in the treatment of obesity. The action of gastroenterology associations and scientific working groups can make an important contribution to the management of the obesity global pandemic. For this, preparation of guidelines on obesity treatment and management of obesity-related complications, hands-on training on endoscopic treatment methods, research for the development of gastroenterological treatments, establishment of obesity departments in gastroenterology clinics, and fellowship practice trainings for obesity can be provided. We believe that increasing awareness among gastroenterologists about their privileged position in the management of obesity treatment, bariatric surgery complications, and related diseases will enable them to use their skills effectively.

## Figures and Tables

**Table 1.** Demographic Characteristics of the Participants

	Total (n= 117)
Female/male	37/80
Age category n(%)	3
<30 years	3 (2.6)
30-40 years	42 (35.9)
40-50 years	44 (37.6)
50-60 years	18 (15.4)
60-70 years	9 (7.7)
>70 years	1 (0.9)
Years of professional experience (%)	3
1-5	44 (37.6)
6-10	20 (17.1)
11-15	17 (14.5)
16-20	13 (11.1)
>20	23 (19.7)

Parameters are expressed as n (%).

**Table 2.** Perception of Participants About Obesity

	Mean	1	2	3	4	5	1-2	4-5	*P*
Obesity is a chronic disease	4.45 ± 0.73	0 (0)	5 (4.3)	2 (1.7)	45 (38.5)	65 (55.6)	4.3	94.1	<.001
Obese patients are more difficult to treat than normal-weight patients	3.87 ± 0.82	0 (0)	11 (9.4)	15 (12.8)	69 (59)	22 (18.8)	9.4	77.8	<.001
I have negative reactions about the appearance of obese patients	2.44 ± 1.07	25 (21.4)	41 (35)	27 (23.1)	22 (18.8)	2 (1.7)	56.4	20.6	<.001
Obesity complicates the management of gastroenterological diseases	4.07 ± 0.73	0 (0)	7 (6)	6 (5.1)	75 (64.1)	29 (24.8)	6	88.9	<.001
Obesity is a negative prognostic factor forthe gastroenterological diseases	4.36 ± 0.59	0 (0)	1 (0.9)	4 (3.4)	63 (53.8)	49 (41.9)	0.4	95.7	<.001
Gastroenterologists should be involved in obesity treatment	4.32 ± 0.8	1 (0.9)	3 (2.6)	10 (8.5)	46 (39.3)	57 (48.7)	3.5	88	<.001
I believe that obese patients should be educated about the health risks of obesity	4.03 ± 1.43	0 (0)	0 (0)	2 (1.7)	35 (29.9)	80 (68.4)	0	98.3	<.001
Obese patients lose a significant amount of weight with my treatment methods	3.41 ± 0.81	1 (0.9)	13 (11.1)	47 (40.2)	48 (41)	8 (6.8)	12	47.8	<.001
For most obese patients, long-term maintenance of weight loss is impossible	3.46 ± 0.88	3 (2.6)	16 (13.7)	28 (23.9)	64 (54.7)	6 (5.1)	16.3	59.8	<.001

Answer categories: 1, strongly disagree; 2, disagree; 3, neutral; 4, agree; 5 strongly agree. Parameters are expressed as mean with SD and n (%).

**Table 3.** Conditions Considered as a Cause of Obesity by the Participants

	Mean	1	2	3	4	5	1-2	4-5	*P*
Income status	3.03 ± 1.09	14 (12)	22 (18.8)	37 (31.6)	36 (30.6)	8 (6.8)	30.8	37.4	.270
Educational status	3.37 ± 1.18	12 (10.3)	12 (10.3)	33 (28.2)	40 (34.2)	20 (17.1)	20.6	51.3	<.001
Physical inactivity	4.61 ± 0.75	1 (0.9)	2 (1.7)	7 (6)	21 (17.9)	86 (73.5)	2.6	91.5	<.001
Overeating	4.49 ± 0.72	1 (0.9)	1 (0.9)	7(6)	38 (32.5)	70 (59.8)	1.8	92.3	<.001
High-fat diet	4.25 ± 0.88	1 (0.9)	3 (2.6)	19 (16.2)	36 (30.8)	58 (49.6)	3.5	80.4	<.001
Genetic factors	3.68 ± 0.97	3 (2.6)	10 (8.5)	32 (27.4)	48 (41)	24 (20.5)	11.1	61.5	<.001
Inadequate nutritional information	3.79 ± 1.03	5 (4.3)	8 (6.8)	22 (18.8)	53 (45.3)	29 (24.8)	11.3	70.1	<.001
Psychological problems	3.92 ± 0.92	2 (1.7)	6 (5.1)	25 (21.4)	50 (42.7)	34 (29.1)	6.8	71.8	<.001
Lack of willpower	4.11 ± 0.89	1 (0.9)	4 (3.4)	22 (18.8)	43 (36.8)	47 (40.2)	4.3	77	<.001
Metabolic disorder	3.89 ± 0.98	1 (0.9)	10 (8.5)	27 (23.1)	41 (35)	38 (32.5)	9.4	67.5	<.001
Endocrine disorder	3.71 ± 1.14	5 (4.3)	13 (11.1)	28 (23.9)	35 (29.9)	36 (30.8)	15.4	60.7	<.001
Gut microbiota	3.13 ± 1.02	6 (5.1)	26 (22.2)	41 (35)	34 (29.1)	10 (8.5)	27.3	37.6	.094
Genetic disorder	3.43 ± 1.12	6 (5.1)	20 (17.1)	29 (24.8)	41 (35)	21 (17.9)	22.2	52.9	<.001

Answer categories: 1, not important; 2, somewhat important; 3, moderately important; 4, important; 5 very important. Parameters are expressed as mean with SD and n(%).

**Table 4.** Factors Preventing Gastroenterologists from Participating in Weight Management of Obese Patients

	Mean	1	2	3	4	5	1-2	4-5	*P*
Obesity treatment is not in my area of interest	2.33 ± 1.81	38 (32.5)	29 (24.8)	26 (22.2)	21 (17.9)	3 (2.6)	57.3	20.5	<.001
I do not have enough time	3.09 ± 1.37	19 (16.2)	25 (21.4)	22 (18.8)	28 (23.9)	23 (19.7)	37.6	43.6	.351
I do not find treatment options effective	2.58 ± 1.23	31 (26.5)	23 (19.7)	34 (29.1)	22 (18.8)	7 (6)	46.2	24.8	.793

Answer categories: 1, not important; 2, somewhat important; 3, moderately important; 4, important; 5, very important. Parameters were expressed as mean with SD and n (%).

**Table 5.** Obesity Treatment Was Considered Effective for Which GI Diseases

	Mean	1	2	3	4	5	1-2	4-5	*P*
GERD	3.70 ± 1.39	14 (12)	11 (9.4)	18 (15.4)	27 (23.1)	47 (40.2)	21.4	63.3	<.001
Functional dyspepsia	3.22 ± 1.09	11 (9.4)	15 (12.8)	39 (33.3)	41 (35)	11 (9.4)	22.2	44.4	<.001
IBS	2.99 ± 1.10	12 (10.3)	26 (22.2)	39 (33.3)	31 (26.5)	9 (7.7)	32.5	34.2	.782
IBD	2.28 ± 1.05	30 (25.6)	44 (37.6)	26 (22.2)	14 (12)	3 (2.6)	63.2	14.6	<.001
NAFLD	4.41 ± 1.03	5 (4.3)	1 (0.9)	15 (12.8)	16 (13.7)	80 (68.4)	5.2	82.1	<.001
GI cancers	3.61 ± 1.25	9 (7.7)	14 (12)	27 (23.1)	30 (25.6)	37 (31.6)	19.7	57.2	<.001

Answer categories: 1, not important; 2, somewhat important; 3, moderately important; 4, important; 5, very important. Parameters were expressed as mean with SD andn (%).

GERD, gastroesophageal reflux disesase; GI, gastrointestinal; IBD, inflammatory bowel disease; IBS, irritable bowel syndrome; NAFLD, nonalcoholic fatty liver disease.
